# Prediction of the Non-Reducing Biomineralization of Nuclide–Microbial Interactions by Machine Learning: The Case of Uranium and *Bacillus subtilis*

**DOI:** 10.3390/toxics13040305

**Published:** 2025-04-13

**Authors:** Shirong Qiang, Leijin Liu, Siqi Li, Shuang Wang, Xinyang Huang, Jiaxin Yang, Jiayu Song, Yue Zhang, Yongxiang Huang, Qiaohui Fan

**Affiliations:** 1 Key Laboratory of Preclinical Study for New Drugs of Gansu Province, Institute of Physiology, School of Basic Medical Sciences, Lanzhou University, Lanzhou 730000, China; 2 School of Stomatology, Lanzhou University, Lanzhou 730000, China; 3 Northwest Institute of Eco-Environment and Resources, Chinese Academy of Sciences, Lanzhou 730000, China; 4 The First School of Clinical Medicine, Lanzhou University, Lanzhou 730000, China

**Keywords:** uranium, *Bacillus subtilis*, machine learning, non-reducing biomineralization

## Abstract

*Bacillus subtilis* exhibits a great affinity to soluble U(VI) through non-reducing biomineralization. The pH value, temperature, initial uranium concentration, bacterial concentration, and adsorption time are recognized as the five environmental sensitive factors that can regulate the degree of non-reductive biomineralization. Most of the current studies have focused on the regulatory mechanisms of these factors on uranium non-reductive mineralization. However, there are still few reports on the importance of these factors in influencing non-reductive mineralization, as well as on how to regulate these factors to increase the efficiency of non-reductive mineralization and enhance the enrichment of *Bacillus subtilis* on uranium. In this work, a deep learning neural network model was constructed to effectively predict the effects of changes in these five environmental sensitivity factors on the non-reducing mineralization of *Bacillus subtilis* to uranium. Accuracy (99.6%) and R^2^ (up to 0.89) confirm a high degree of agreement between the predicted output and the observed values. Sensitivity analysis shows that in this model, pH value is the most important influencing factor. However, under different pH values, temperature, initial uranium concentration, adsorption time, and bacterial concentration have different effects. When the pH value is lower than 6, the most important factor is temperature, and once the pH value is greater than 6, the initial concentration is the most important factor. The results are expected to provide a theoretical basis for regulating the enrichment degree of U(VI) by *Bacillus subtilis*, achieving the maximum long-term stable fixation of U(VI), and understanding the environmental chemical behavior of uranium under different conditions.

## 1. Introduction

Uranium (U) is one of the radioactive chemical elements of iii. b actinides. It is the natural element with the largest atomic number and relative atomic mass, possessing both chemical toxicity and radiation toxicity. With the development of nuclear energy and nuclear tests, the occurrence of nuclear accidents, and the continuous development of the uranium mining and smelting industry, the soil pollution and health problems caused by uranium are increasing [1,2,3]. Understanding the environmental processes such as migration, transformation, and cycling of uranium is of great theoretical significance for accurately assessing the environmental effects of uranium and for the prevention and remediation of pollution. Once it enters the environmental system, uranium can undergo physical, chemical, and biochemical reactions such as adsorption/desorption, precipitation/dissolution, oxidation/reduction, biological absorption/release, and biomineralization with environmental media. These complex reactions and process controls influence the migration, transformation, circulation, and return of uranium in the environment; therefore, the microenvironmental chemical behavior of uranium has always been the focus and frontier of environmental radiochemistry [4,5]. The valences of uranium are III, IV, V, and VI, respectively [6], with U(IV) and U(VI) being the most common valence states. U(IV) usually exists in the form of low-toxic deposition compounds with high stability and low solubility, whereas U(VI) usually exists as UO_2_^2+^, with high solubility and mobility.

Microorganisms play an extremely important role in regulating the environmental behavior of elements. The interaction mechanisms between microorganisms and uranium include bioadsorption, biomineralization, and biotransformation. After uranium undergoes biomineralization, stable products can be formed, and its mobility in the soil can be reduced. The highest abundance of microorganisms in uranium tailings soil is *Bacillus subtilis*, and it has good uranium tolerance and enrichment. As a kind of Gram-positive aerobic bacteria widely distributed in soil and on surfaces, *Bacillus subtilis* shows a great affinity to soluble U(VI) [7] through non-reducing biomineralization [8,9]. Compared with the reductive biomineralization that occurs under anaerobic conditions, non-reducing biomineralization directs fixation of uranium in the form of stable minerals, in which U(VI) does not undergo reduction. In uranium pollution control, the non-reductive mineralization by *Bacillus subtilis* primarily relies on the interaction between its surface functional groups and uranium. The entire process does not involve the reduction of uranium; hence, it is referred to as non-reductive mineralization. This process has great application prospects in the long-term stable fixation of uranium and is very important for controlling uranium pollution and understanding the migration and transformation processes of uranium in the environment. The pH value, temperature, initial uranium concentration, bacterial concentration, and adsorption time are recognized as the five factors that can regulate the degree of non-reductive mineralization environmental sensitivity factors. Most of the current studies have focused on the regulatory mechanisms of these factors on uranium non-reductive mineralization. However, there are still few reports on the importance of these factors in influencing non-reductive mineralization, as well as on how to regulate these factors to increase the efficiency of non-reductive mineralization and enhance the enrichment of *Bacillus subtilis* on uranium, thereby maximizing the long-term stable fixation of U(VI).

A deep learning neural network (DLNN) is a supervised automatic learning machine learning algorithm using a differentiated neural network composed of multilayer nodes to solve complicated problems [10,11]. A neural network has a particular compute function in each layer, using different weight matrices to connect different layers, provide neural network input and calculation, and ultimately output corresponding results [12,13]. Common neural network models include convolutional neural networks (CNNs), stacked long and short-term memory (LSTM), recurrent neural networks (RNNs), and generative countermeasure networks (GANs), etc. [14,15,16,17,18,19]. Neural networks can simulate the complex functions of the human brain and be used for nonlinear data and problems like decision-making, diagnosis, prediction, and control [20,21,22,23]. Common DLNN models are used for image recognition, speech recognition, and even geological disaster prediction [24,25,26]. The advantages of the deep learning neural network model are mainly manifested in the following three aspects: (1) Due to the “deep” feature of deep learning neural networks, they can capture complex relationships between data, enabling them to better grasp and analyze data information and obtain meaningful conclusions from data [25,27,28]. (2) Deep learning neural networks can extract effective features from data, thereby reducing engineering characterized by manual operation and the work needing multiple cross-validations in many artificial engineering tasks [25,29,30]. (3) Deep learning neural networks do not need to rely on specific content or algorithms. They can automatically adapt to the simulated world through a large number of samples. If effectively trained, they can solve many nonlinear problems [31,32].

In this article, we have constructed a DLNN neural network model based on the experimental data collected, aiming to predict the influence degree of five environmental sensitivity factors on the non-reductive mineralization of *Bacillus subtilis* to uranium, the result is expected to provide a theoretical basis for regulating the enrichment degree of *Bacillus subtilis* for U(VI), achieving the maximum long-term stable fixation of U(VI), and understanding the environmental chemical behavior of uranium under different conditions.

## 2. Method

### 2.1. Data Extraction and Dataset Construction

To better enable the computer to read data, some variables in the database are converted into numbers. The data of each model are randomly divided into two datasets: the training set and the verification set. The former is used to build the neural network model, and the latter is used to check the accuracy of the model. The five independent variables of temperature, pH, initial concentration (U), time, and biosorbent concentration were introduced, and the adsorption rate of uranium was used as a variable. Among them, the variable value distribution of biosorbent concentration is relatively concentrated, and the differences are small. Time, pH, and temperature variables value distribution gradually increase, and the variable value distribution of the initial concentration (U) is relatively scattered. The specific distribution features of the data are shown in Table 1.

### 2.2. Fully Connected Deep Neuron Network (DNN)

In contrast to the conventional single-layer perceptron, the deep neural network DNN is characterized by an intricate operational interplay between each node within a given layer and all nodes in the immediately subsequent layer, a feature that justifies the term fully connected within its nomenclature. The canonical structure of a DNN is tripartite, comprising the initial layer designated as the input layer, the terminal layer earmarked for output, and the intermediate layer, which is colloquially termed the hidden layer. It is not uncommon for fully connected neural networks to encompass an array of hidden layers, which serve to augment the networks capacity to delineate and isolate the nuanced characteristics inherent in the dataset. Within the purview of our scholarly endeavor, we have endeavored to construct a sophisticated deep learning neural network model using “AI for Science produced” by Beijing Diji Tech. This is a platform for speeding up the implementation and explanation of deep-learning algorithms, the objective of which is to prognosticate the adsorptive affinity of uranium by the microorganism *Bacillus subtilis*.

The model consists of one input layer, four hidden layers, and one output layer, with each hidden layer containing 95 neurons. The five influencing factors, the pH, time, temperature, initial concentration of uranium, and biosorbent concentration, are entered into the input layer and then processed and analyzed by the hidden layers. After feature transformation into the abstract features, the output layer obtains the classification results. In each neuron of the hidden layer, the rectified linear unit (ReLU) activation function was applied to the model, and the data were finally transformed to the output layer by the transfer sigmoid transfer function. Finally, this study uses the adaptive moment estimation (Adam) algorithm to construct a classification boundary to train the deep neural network model. This algorithm can help achieve a fast rate of convergence and good classification performance.

The architecture of the deep neural network (DNN) is fundamentally linear, employing activation functions to address challenges that cannot be resolved by nonlinear models. Z = ∑ω_i_x_i_ + b, the coefficient matrix of linear relations, denoted as ω, along with the bias vector b, are employed to perform a series of linear operations on the input value vector x. Subsequently, an appropriate activation function is selected for the activation processes. The activation functions utilized in this study include the logarithmic Sigmoid, ReLU, and Tanh functions. Furthermore, each layer of the network is characterized as dense.

$$\mathrm{Sigmoid}:f\left(x\right)=\frac{1}{1+e^{-x}}$$

$$\mathrm{Relu}:f\left(x\right)=\left\{\begin{array}{c} -x,\;if\;x\geq 0\\ o,\;if\;x<0\; \end{array}\right.$$

$$\mathrm{Tan}h:\;f\left(x\right)=\frac{e^{x}-e^{-x}}{e^{x}+e^{-x}}$$

where x represents the input signal to the neuron and f denotes the activation function.

### 2.3. Model Validation Analysis

The collected database was randomly partitioned into a training dataset comprising 60% of the total data and a validating dataset consisting of the remaining 40%. These datasets were utilized for model training and validation, respectively. To assess the model’s performance, we calculated the accuracy by comparing the predicted outputs from the model with the actual values in the validation set.

$$Accuracy=\frac{N^{\prime }}{N}\times 100\%$$

**Figure 1:**
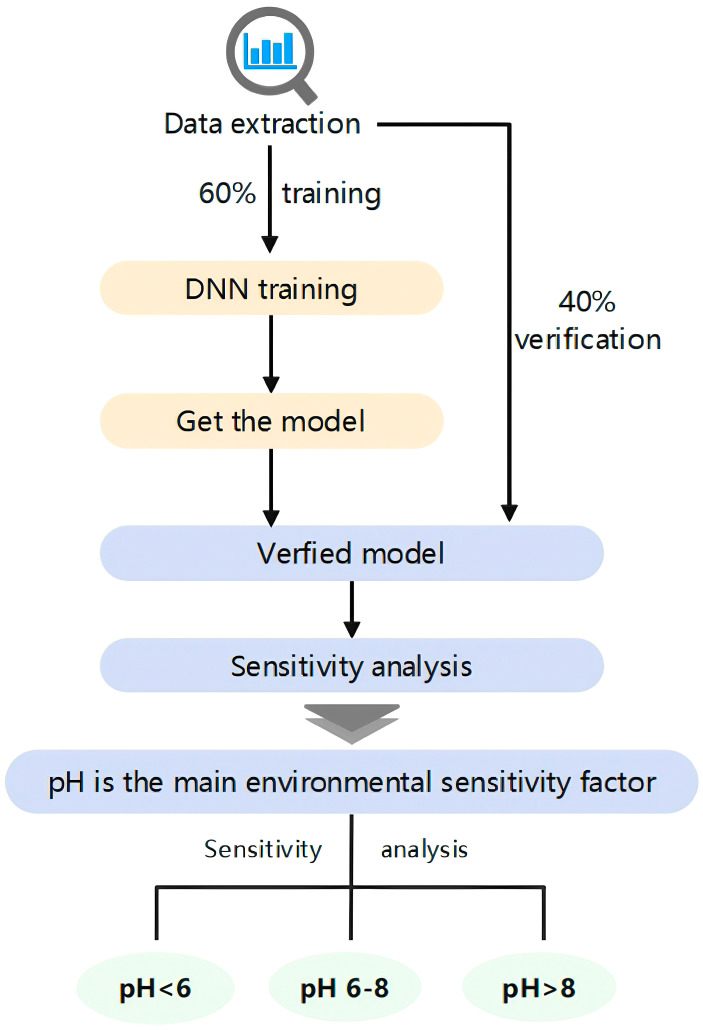
The experimental flow chart.

In this context, N denotes the total number of samples in the database while N′ representing the count of samples for which the predicted outputs align with the actual values found in the validation dataset.

$${\;R}^{2}=1-\frac{\sum_{i=1}^{n}{\left(y_{i}-\bar{x}\right)}^{2}}{\sum_{i=1}^{n}{\left(x_{i}-\bar{x}\right)}^{2}}$$

where x_i_ represents the actual value, $y_{i}$ is the forecasted value, and $\bar{x}$ denotes the mean of x. The statistic R^2^ is used to assess the variance between the predicted and actual values regarding uranium adsorption by *Bacillus subtilis*, serving as a metric for evaluating the accuracy of quantitative models (Figure 1).

### 2.4. Loss Function

In this analysis, the mean squared error (MSE), a widely used loss function, is utilized to illustrate the model’s training progression and to quantify the discrepancy between predicted and actual values. MSE provides a straightforward method for calculating the “mean error”. A lower MSE indicates a more accurate prediction model in representing the experimental results.

$$MSE=\frac{1}{n}\sum_{i=1}^{n}{\left(y_{i}-{\hat{y}}_{i}\right)}^{2}$$

where y_i_ is the real data, ${\hat{y}}_{i}$ is the fitted data, and n is the number of samples.

To gain a deeper understanding of how independent variables influence bacterial-like types, we employed a deep neural network (DNN) model for training. During the training process, we leveraged a loss function to evaluate the model’s error and continuously optimized its parameters. After numerous iterations and adjustments, we observed that the loss function value gradually decreased and stabilized after approximately 240 iterations, demonstrating the model’s high accuracy. Next, we further developed quantitative models to analyze the extent of bacterial-like activity and presented the learning processes of four DNN-based machine learning models along with the changes in their loss functions. The loss function values of these models rapidly declined as the number of iterations increased, indicating a continuous improvement in model accuracy. Finally, we conducted a sensitivity analysis of the models using the Shapley additive explanations (SHAP) method.

### 2.5. Sensitivity Analysis

The sensitivity analysis of the model was determined by the Shapley additive explanation (SHAP) [33].

It embodies the potential efficacy of the input data with respect to the output. In a neural network, the activation level of each neuron is contrasted against a reference value, and scores are allocated in accordance with the contribution, thereby deducing the input’s contribution. The activation degree of each neuron within the neural network is compared to a reference value, and the contribution is inferred by assigning scores based on that contribution. The model is capable of generating predicted values that correspond precisely to the samples. Moreover, the SHAP value represents the contribution proportion of such features in each sample.

Supposing that the nth feature of the sample is X_in_, the model predicts yi of the ith sample, and the baseline of the entire model (ordinarily the arithmetic mean of the target variable of all samples) is Y_base_, then the SHAP value adheres to the following formula:

$$Y_{i}=Y_{\mathrm{base}}+f\left(X_{i1}\right)+f\left(X_{i2}\right)+\cdots +f\left(X_{in}\right)$$

**Figure 2:**
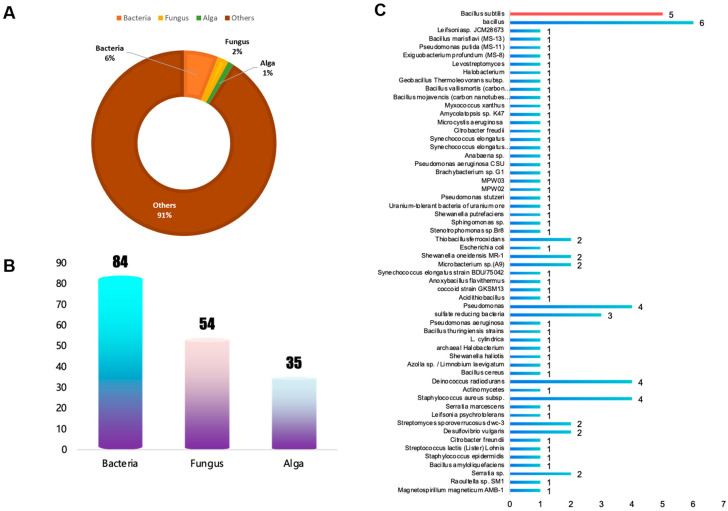
The characteristics of articles related to uranium adsorption: (**A**) percentage, (**B**) specific number, (**C**) the species of bacteria, red bar represents the Bacillus subtilis.

Intuitively, $f\left(X_{in}\right)$ is the contribution of the nth feature in the ith sample to the final predicted value $Y_{i}$. The sensitivity ranking of the independent variables is illustrated in Figure 2C.

### 2.6. Statistical Analysis

All statistical analyses were conducted using R software (R 4.2.3) and Excel (2019). One-way analysis of variance (ANOVA) was used to compare among multiple groups.

## 3. Results

### 3.1. Construction of a Database for Machine Learning

In this study, 14,936 articles related to uranium adsorption and mineralization were retrieved from PubMed and ScienceDirect using “uranium adsorption mineralization” as the keywords, and the publication time was limited to 29 February 2024 (Figure 2A). Among them, 598 documents (accounting for about 6%) use bacteria for uranium adsorption and mineralization, followed by fungi and algae, accounting for about 2% and 1%, respectively (Figure 2A,B). This shows that as a new type of uranium adsorbent, bacteria in microorganisms are widely studied by researchers [34].

There are 58 kinds of bacteria involved, of which the largest number of Bacillus-related species was 14. In the end, the data related to Bacillus (a total of 133) were used as a database for model training and verification (Figure 2B,C).

### 3.2. Construction of a DLNN Model for Uranium Adsorption by Bacillus subtilis

**Figure 3:**
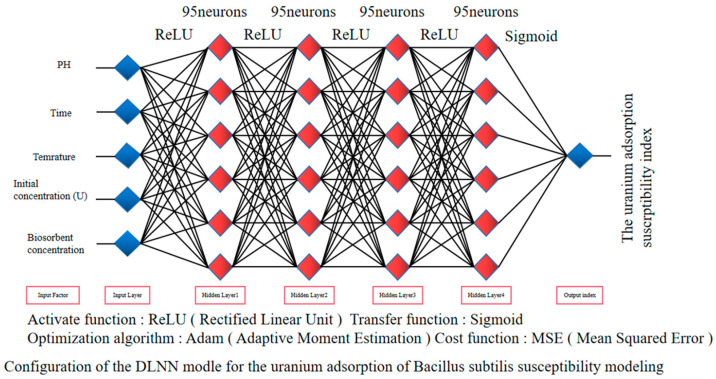
Configuration of the DLNN model for the uranium adsorption of *Bacillus subtilis* susceptibility modeling.

We established a deep learning neural network (DLNN) model consisting of one input layer, four hidden layers, and one output layer, with each hidden layer containing 95 neurons. The five influencing factors, the pH, time, temperature, initial concentration of uranium, and biosorbent concentration, are entered into the input layer and then processed and analyzed by the hidden layers. After feature transformation into the abstract features, the output layer obtains the classification results. In each neuron of the hidden layer, the rectified linear unit (ReLU) activation function was applied to the model, and the data were finally transformed to the output layer by the transfer sigmoid transfer function. Finally, this study uses the adaptive moment estimation (Adam) algorithm to construct a classification boundary to train the deep neural network model. This algorithm can help achieve a fast rate of convergence and good classification performance (Figure 3).

### 3.3. The Evaluation of the Model Prediction Performance

**Figure 4:**
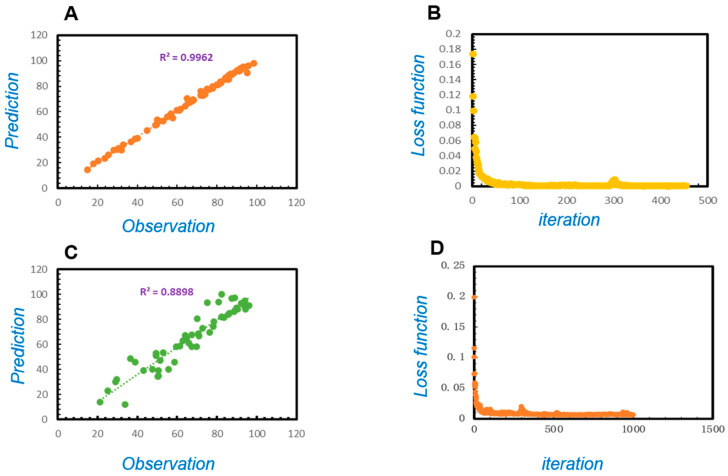
The tests of the model prediction performance: (**A**) the R^2^ of the training set, (**B**) loss function of the training set, (**C**) the R^2^ of the validating set, and (**D**) loss function of the validating set.

The R^2^ values were used to describe the model performance for prediction ability. The results show, R^2^ = 0.9962 between the training set observation results and prediction results (Figure 4A), while R^2^ = 0.8898 between the validating set predictive values and observed values (Figure 4C), which revealed that the model for uranium adsorption by *Bacillus subtilis* demonstrated good predictive performance.

To explore the effect of independent variables on the uranium adsorption rate and mineralization efficiency, a DNN-based model was trained with an optimization process that required a loss function to calculate the error. The training process, with decreasing loss function, was shown following increasing parameter adjustment and iteration. The loss functions of the training set and the validating set were shown by the corresponding learning processes (Figure 4B,D).

### 3.4. Sensitivity Analysis of the Model

**Figure 5:**
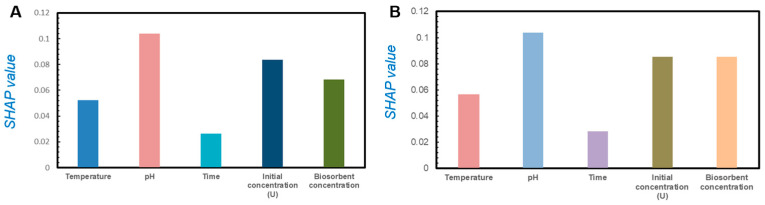
The contribution of variables: (**A**) the bar of absolute SHAP values of the training set, (**B**) the bar of absolute SHAP values of the validating set.

We tried to analyze the effect of independent variables on output dependent variables. The sensitivity of the model was analyzed using the SHAP method. Figure 5 shows the sensitivity of the SHAP analysis of five variables in the classification model of the training set (Figure 5A) and the validating set (Figure 5B), including the temperature, pH value, initial uranium concentration, adsorption time, and bacterial concentration. It was observed that the pH played the most important role in the non-reductive mineralization process of uranium by *Bacillus subtilis*, followed by the initial uranium concentration, temperature, and bacterial concentration. The importance of adsorption time is the lowest.

### 3.5. Sensitivity Analysis of the Model at Different pH Values

**Figure 6:**
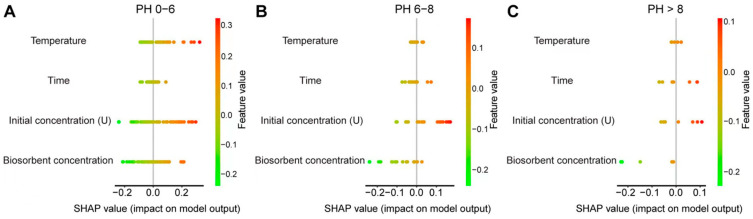
The specific potential increased/decreased effects of the data on the activities at (**A**) pH values of 0 to 6 (training), (**B**) pH values of 6 to 8, and (**C**) pH greater than 8 (training).

We combined the prediction set with the data from the training set for analysis. Figure 6 indicates the potential increased (red points) or decreased (green points) effects of the data on the activities. As can be seen from Figure 6A, the data show that under pH values of 0 to 6, temperature has the greatest impact, followed by initial concentration (U), biosorbent concentration, and time. Figure 6B shows that under pH values of 6 to 8, initial concentration (U) has the greatest impact, followed by time, then temperature, and biosorbent concentration has the least impact. The order of factors affecting uranium non-reductive mineralization at a pH greater than 8 is the same as that at pH 6–8 (Figure 6C).

## 4. Discussions

### 4.1. A DLNN Neural Network Model with Good Predictive Performance Is Constructed

In order to predict the non-reducing mineralization ability of uranium to *Bacillus subtilis*, we constructed a DLNN neural network model. The process of the non-reductive mineralization is as follows: (1) Adsorption stage: *Bacillus subtilis* first adsorbs uranium onto its surface through functional groups such as amino, carboxyl, and phosphate groups. (2) Mineralization stage: The adsorbed uranium then gradually transfers into the bacterial cell in the form of amorphous particles and eventually forms stable autunite ((NH_4_)(UO_2_)(PO_4_)·3H_2_O), thus becoming fixed. Therefore, we measure the non-reductive mineralization ability of *Bacillus subtilis* toward uranium by determining its adsorption capacity for uranium. The stronger the adsorption capacity, the stronger the ability of non-reducing mineralization for *Bacillus sutilis*; the weaker the adsorption capacity, the weaker the ability is. After building the model, we validated it with R^2^ and loss values. The closer the R^2^ value is to 1, the better the prediction performance. The R^2^ value of the prediction set of this model is 0.8898 (Figure 4C), indicating that the model has good prediction performance. The loss value essentially refers to the difference between the predicted value and the true value of the model. The smaller the difference, the closer the loss value is to 0, indicating that the predictive performance of the model is better. It was observed that in the validating set, the loss function rapidly declined with the increase in iteration, demonstrating that the accuracy of the models consistently improved (Figure 4D). In summary, the DLNN model we constructed has good predictive performance.

### 4.2. pH Value Is the Main Factor Affecting Non-Reducing Uranium Mineralization

In order to clarify the most important factors affecting the non-reducing mineralization of uranium on *Bacillus subtilis*, we conducted sensitivity analyses on the data of the prediction and training sets (Figure 5). The results showed that the pH value was the most significant factor affecting both the training and prediction sets. Song et al. (2019) used XPS to investigate the composition and valence state of uranium–*Bacillus subtilis* bio-minerals, revealing that the product remains hexavalent [35]. This suggests that the immobilization of uranium by *Bacillus subtilis* is non-reductive, and the affinity between uranium and *Bacillus subtilis* is primarily driven by electrostatic and complexation interactions. In acidic pH, U(VI) in aqueous solution primarily exists as UO_2_^2+^. As the pH increases, negatively charged uranyl ions such as (UO_2_)_3_(OH)_7_^−^ and UO_2_(OH)^3−^ dominate the solution. When the pH further increases to alkaline conditions, U(VI) precipitates as insoluble uranate precipitates (Na_2_U_2_O_7_) [25]. For *Bacillus subtilis*, changes in pH mainly impact its surface charge properties. Experiments have shown that as the pH increases, the zeta potential on the bacterial surface changes from positive to negative. When the pH is below 4, the zeta potential on the bacterial surface is >0, while above 4, it becomes negative. Thus, with increasing pH, negative charges in the solution increase, leading to an increase in repulsive forces between bacteria and uranium. Compared to other factors, pH directly alters the chemical form of uranium and the charge on the bacterial surface, thereby directly impacting their non-reductive mineralization process. Therefore, we speculate that, under similar conditions, in acidic soils rich in *Bacillus subtilis*, the non-reductive mineralization degree of uranium will be higher, and its mobility will be lower. Moreover, we suggest that when using *Bacillus subtilis* to treat uranium contamination, the pH value situation should be the first concern. Acidic conditions will be more conducive to the enrichment of uranium.

### 4.3. Sensitivity Analysis Under Different pH Values

Since pH is the most important factor affecting the non-reductive mineralization of uranium, we then discussed the influence of temperature, time, initial concentration, and biosorbent concentration on uranium adsorption under different pH conditions. The results showed that when the pH was acidic (pH 0–6), apart from the initial concentration (U), temperature showed an absolute superiority, followed by biosorbent concentration and time (Figure 6A). This may be because uranium is ionic under acidic conditions, and the higher the temperature, the more ions are dissociated. The increased temperature accelerates the Brownian motion of U(VI) ions, thereby increasing the adsorption capacity. The enrichment of uranium by *Bacillus subtilis* is an endothermic process. Within a certain range, the higher the temperature, the higher the Gibbs free energy, and the more obvious the mineralization reaction and the higher the degree of mineralization. However, when the pH is greater than 6, the influence of temperature on the non-reductive mineralization has been significantly reduced. Under such conditions, the initial concentration (U) has the greatest impact, followed by time, then temperature, and biosorbent concentration has the least impact (Figure 6B,C). We suggest this may be due to the negatively charged uranyl ions and the U(VI) precipitates increasing when the pH is greater than 6. At this time, even if the temperature rises, it will not increase the electrostatic interaction and complexation between uranium and *Bacillus subtilis* cell membranes.

Overall, the initial concentration is the second factor that has a significant impact on the non-reductive mineralization of uranium by *Bacillus subtilis* (Figure 5). When the number of *Bacillus subtilis* is sufficient, there are more adsorption sites on its cell surface. Therefore, the higher the initial uranium concentration and the higher the ionic concentration, the easier it is for the bacteria to occupy the adsorption sites, thereby increasing the reaction efficiency. When pH < 6, since most uranium exists in ionic form, although the initial concentration is too high, it will increase the enrichment degree of uranium by the bacteria, but the exposure of uranium to the bacteria will also cause certain toxicity, thereby reducing the reaction efficiency. Under this condition, the influence of temperature on non-reductive mineralization is slightly greater than the initial concentration. When pH > 6, there is less ionic uranium, and a relatively larger number of *Bacillus subtilis* can provide sufficient adsorption sites. At this time, the influence of the initial concentration on non-reductive mineralization takes the leading position. Although the influence of time and bacterial quantity on non-reductive mineralization is relatively small compared to the other three factors, the sensitivity ranking under different pH conditions is also different. We speculate that the possible reasons are as follows: when pH < 6, since uranium is in an ionic state, the more bacteria means the more adsorption sites, and the time required for the reaction to reach equilibrium is certain. At this time, the influence of bacterial quantity on the degree of mineralization is relatively large; when pH > 6, although there are sufficient adsorption sites due to the low ionic concentration, the influence on mineralization is not significant. By extending the reaction time, more free uranium ions can occupy the adsorption sites, so in this condition, the influence of time is greater than that of bacterial quantity.

In conclusion, we speculate that the non-reductive mineralization of uranium in slightly acidic soil will be more pronounced. Moreover, this phenomenon will be more obvious in the vicinity of the equator and during hot seasons. Compared to other regions and times, uranium is more fixed and less likely to migrate; while pollution occurs in neutral or slightly alkaline soil, the migration rate of uranium depends on the number of *Bacillus subtilis* in the soil. In addition, when treating uranium pollution with *Bacillus subtilis*, the culture temperature should be as high as possible in acidic conditions to obtain the best enrichment effect.

## 5. Conclusions

In this study, we introduced five environmental sensitivity factors on the non-reducing mineralization for *Bacillus subtilis* of uranium, including temperature, pH value, initial uranium concentration, adsorption time, and bacterial concentration. Then, we analyzed and fit the data obtained from the database through DLNN. We have successfully established a model that can be used to predict the effects of changes in environmental sensitivity factors on the non-reducing mineralization for *Bacillus subtilis* of uranium. The training verification result of the model shows that this model can effectively predict the non-reducing mineralization characteristics of uranium. Sensitivity analyses show that in this model, the pH value is the most important factor, followed by the initial uranium concentration, temperature, and bacterial concentration. The importance of adsorption time is the lowest. Therefore, under similar conditions, in the slightly acidic soil rich in *Bacillus subtilis*, the non-reducing mineralization degree of uranium will be higher, and its mobility will be lower. Using *Bacillus subtilis* to treat uranium-contaminated soil under acidic conditions is more conducive to the enrichment of uranium. However, under different pH values, the temperature, initial uranium concentration, adsorption time, and bacterial concentration amount have different effects on the non-reductive mineralization. When the pH value is lower than 6, apart from the initial concentration (U), temperature shows an absolute superiority, followed by biosorbent concentration and time. When the pH is greater than 6, the influence of temperature on the non-reductive mineralization is significantly reduced. Under such conditions, the initial concentration (U) has the greatest impact, followed by time and then temperature, and biosorbent concentration has the least impact. We speculate that if uranium contamination occurs in slightly acidic soil, regions near the equator and hot seasons will have a higher degree of uranium mineralization. Compared to other regions and times, uranium is more stable and less likely to migrate. However, if contamination occurs in neutral or slightly alkaline soil, the migration rate of uranium depends on the number of *Bacillus subtilis* in the soil. In addition, when treating uranium contamination with *Bacillus subtilis*, it is advisable to raise the cultivation temperature as much as possible under acidic conditions to achieve the best enrichment effect. Overall, taking uranium and *Bacillus subtilis* as an example, our study innovatively used machine learning algorithms to calculate the effects of changes in five environmental sensitivity factors on the non-reducing mineralization of *Bacillus subtilis* on uranium and analyze the possible mechanisms. By optimizing and adjusting parameters for different real-world environmental systems, it can make accurate predictions and thus has broad applicability. The research results are expected to provide a theoretical basis for regulating the enrichment degree of U(VI) by *Bacillus subtilis*, achieving the maximum long-term stable fixation of U(VI), and understanding the environmental chemical behavior of uranium under different conditions.

## Figures and Tables

**Table 1:** Characteristics of uranium adsorption model by *Bacillus subtilis*.

Variable	Min	Max	Median	Mean	SD
Temperature/k	283	313	298	300.1128	3.776966
pH	1.3	11	4.9	5.56391	2.319721
Time/h	0.5	3	0.5	1.097744	0.978065
Initial concentration U/(mg/L)	1	300	20	46.10526	70.85008
Biosorbent concentration/(mg/L)	0.125	1.5	0.5	0.582068	0.387437

## Data Availability

The data presented in this study are available on request from the corresponding author.
